# Modified shape index for object-based random forest image classification of agricultural systems using airborne hyperspectral datasets

**DOI:** 10.1371/journal.pone.0213356

**Published:** 2019-03-07

**Authors:** Eric Ariel L. Salas, Sakthi Kumaran Subburayalu

**Affiliations:** Agricultural Research Development Program (ARDP), Central State University, Wilberforce, Ohio, United States of America; University of Wisconsin Milwaukee, UNITED STATES

## Abstract

This paper highlights the importance of optimized shape index for agricultural management system analysis that utilizes the contiguous bands of hyperspectral data to define the gradient of the spectral curve and improve image classification accuracy. Currently, a number of machine learning methods would resort to using averaged spectral information over wide bandwidths resulting in loss of crucial information available in those contiguous bands. The loss of information could mean a drop in the discriminative power when it comes to land cover classes with comparable spectral responses, as in the case of cultivated fields versus fallow lands. In this study, we proposed and tested three new optimized novel algorithms based on Moment Distance Index (MDI) that characterizes the whole shape of the spectral curve. The image classification tests conducted on two publicly available hyperspectral data sets (AVIRIS 1992 Indian Pine and HYDICE Washington DC Mall images) showed the robustness of the optimized algorithms in terms of classification accuracy. We achieved an overall accuracy of 98% and 99% for AVIRIS and HYDICE, respectively. The optimized indices were also time efficient as it avoided the process of band dimension reduction, such as those implemented by several well-known classifiers. Our results showed the potential of optimized shape indices, specifically the Moment Distance Ratio Right/Left (MDRRL), to discriminate between types of tillage (corn-min and corn-notill) and between grass/pasture and grass/trees, tree and grass under object-based random forest approach.

## Introduction

Broadband vegetation indices (VIs) reduce spectral data dimension by limiting the number of bands at different ranges of the electromagnetic spectrum to extract vegetation information from remotely sensed images. Mostly, the bands are selected from the visible and near/mid infrared regions in order to measure the photosynthetic activity of the plant [1] [2], vegetation dynamics [3], biomass abundance [4], predict crop yield [5], and biotic stresses [6]. This reduction of spectral information could pose some drawbacks such as index saturation beyond certain level when estimating high vegetation biomass [7] [8]. Another constraint in the use of existing broadband VIs is the challenge of choosing relevant band centers and widths [9] for agricultural management system mapping, particularly if it involves hyperspectral data where there is increased number of near-continuous bands. Under such circumstances, broadband VIs resort to using only average spectral information over wide widths resulting in loss of crucial information, such as little absorption features caused by the differences of spectral responses from agricultural fields that may be available in those specific narrow bands [10]. These hardly noticeable spectral absorption features could be the key for differentiation of landcover classes with similar spectra, as in the case of crop residue and soil.

Hyperspectral sensors, including the Airborne Visible/Infrared Imaging Spectrometer (AVIRIS) from National Aeronautics and Space Administration (NASA) and Hyperspectral Digital Imagery Collection Experiment (HYDICE) from U.S. Navy Space and Warfare Systems Command, offer improvements in spectral and spatial resolution. Increased number of bands means another challenge for users who need to extract appropriate optimal wavebands for specific applications. As bands become narrow, neighboring bands could have redundant information that may require users to devote more time in data mining and complex processing of removing redundant bands [10] [11]. The potential and challenges of hyperspectral narrowband sensors have inspired the development and application of the shape-based metric called moment distance index (MDI) [12].

MDI was initially developed for the analysis of hyperspectral reflectance curves for vegetation and soil sensitivity studies. Being sensitive to the visible (VIS) to near infrared (NIR) regions where there is a strong difference in the reflectances for vegetation and soil, MDI has been utilized to identify spectral regions for chlorophyll and carotenoids [13], estimate green vegetation fraction [14], detect greenhouses using WorldView-2 and Landsat satellite data [15], discriminate vegetation classes [16] and used as a main component for a new Threshold Relative Radiometric Correction Algorithm (TRRCA) for multiband satellite data [17]. MDI is an effective tool for landcover classification when applied to medium and high spatial resolution images. Aguilar et al. [15] reported MDI as the most important spectral feature to detect vegetative versus non-vegetative regions when tested against other indices such as Normalized Difference Vegetation Index (NDVI), Green Normalized Difference Vegetation Index (GNDVI), Normalized Difference Water Index (NDWI), Enhanced Vegetation Index (EVI), and Plastic-Mulched Landcover Index (PLMI). MDI was superior when used as a component in object-based analysis in discriminating classes of vegetation [16].

The advantage of MDI over other existing spectral VIs for landcover classification is its discriminatory power to characterize the raw shape of the reflectance curve by using all available spectral bands (multispectral or hyperspectral) that could carry additional spectral information useful for vegetation mapping [13]. MDI could also address the challenge of high dimensionality inherent in hyperspectral datasets, by obliterating the need to perform curve transformation (e.g., derivative). Besides, no priori knowledge of optimal wavebands is required to use MDI, nor there is a need to spend much time in removing redundant bands, or aggregating of bands [18], or selecting informative bands [11]. The MDI framework could be explored for optimal use of the spectrum in a computationally simple and broadly applicable manner. Further, image denoising is not required for MDI to work. Even hyperspectral image denoising algorithms cannot guarantee removal of noise from image. In fact, denoising techniques could oversmooth images and leads to information loss [19]. However, the limitation of MDI is that it is an unbounded metric. It increases or decreases as a nontrivial function of the number of bands considered and the shape of the reflectance curve that spans those contiguous bands. This limitation could be an issue when comparing results from different sensors since the spectral resolution could differ from one sensor to another. For example, applying MDI to an AVIRIS image with 220 spectral bands could result differently when using MDI to the HYDICE image with 190 spectral bands. Hence, the main goal of this study is to develop enhanced indices based on the principle of MDI that could ease comparisons across different biome-types and hyperspectral sensors, within the framework of object-based image analysis (OBIA) approach. This study contributes to the (1) improvement in the discrimination between vegetation classes and agricultural management systems by utilizing the potential of the optimized MDIs; and (2) identification of the best combination of variables for image classification using object-based random forest approach.

## Materials and methods

**Fig 1:**
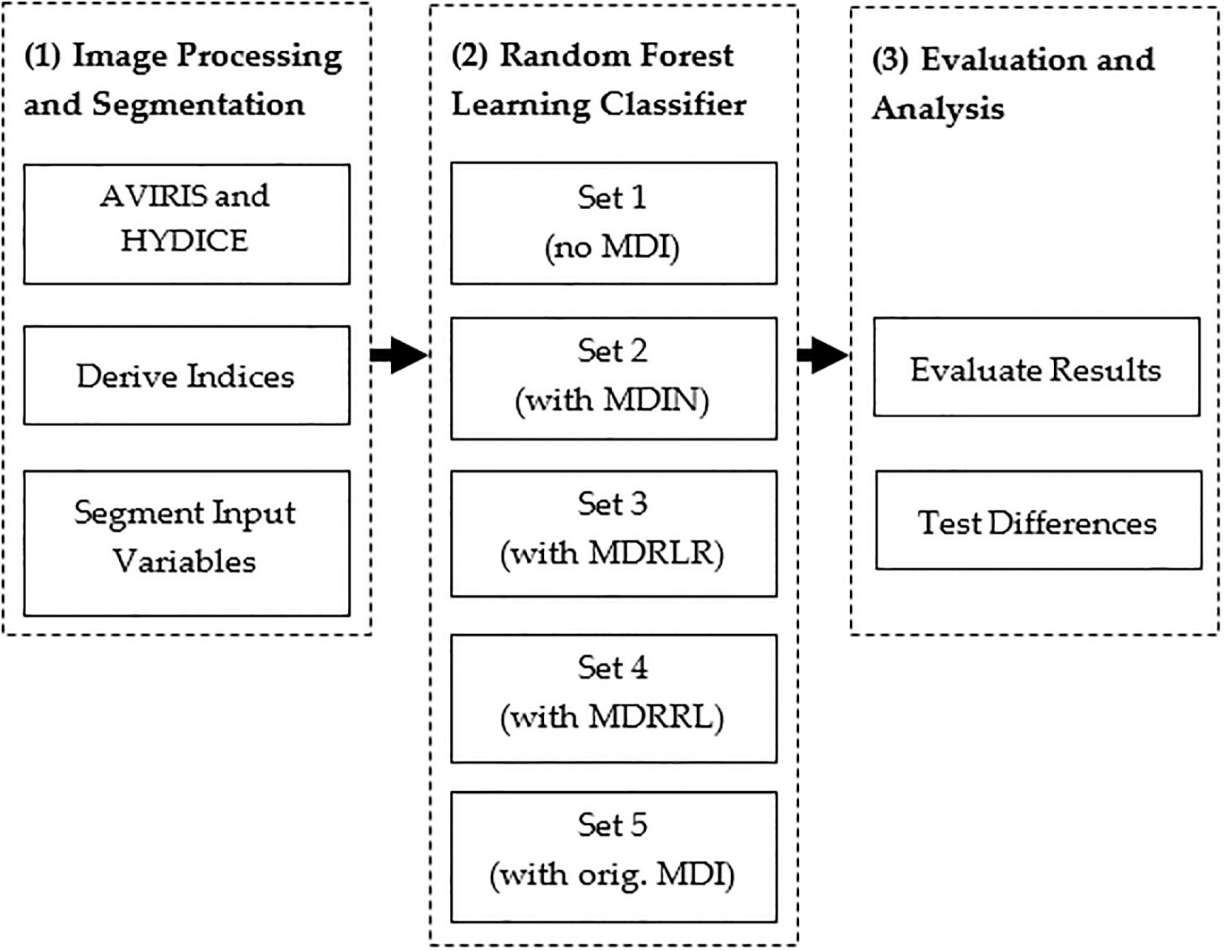
The three major steps used of the study include (1) processing and segmenting the images, (2) applying the random forest classifier, and (3) evaluating and assessing the results.

The methodology was based upon the steps displayed in Fig 1, which included (1) processing and segmenting the images, (2) applying the random forest classifier, and (3) evaluating and assessing the results.

- In the first step, we processed the two airborne image datasets. From the resulting images, we derived spectral indices and textural features. For each derived index and feature, we applied segmentation analysis to produce image objects that served as input variables for our models.
- In the second step, we divided all input variables into different sets. To thoroughly evaluate the classification performance of our new and enhanced indices, we ran different analyses using five sets of data with and without the moment distance metrics. We used each set to run the Random Forest learning classifier.
- In the final step, we evaluated the results from the five sets of data, compared classification accuracies, and checked whether accounting for optimized MDI had improved classification results.

The following provides a summary of data sources, the variables used in the models, model structure, and assessment algorithm.

### Airborne image datasets

**Fig 2:**
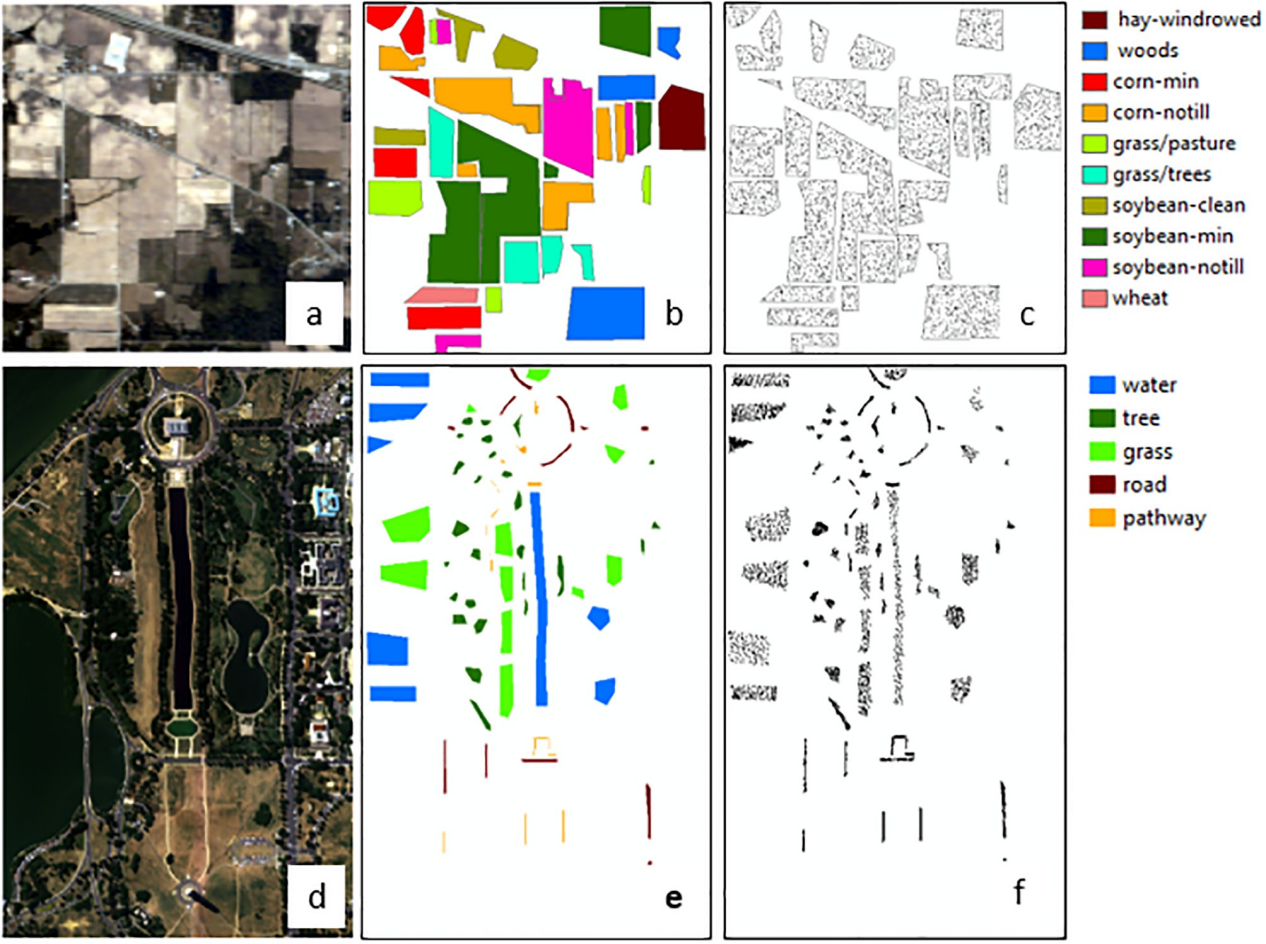
AVIRIS Indian Pines (A) RGB composite image (channels 47, 24, and 14), (B) its reference map and (C) its training and test set. HYDICE Washington DC Mall (D) RGB composite image (channels 51, 41, and 22), (E) its reference map and (F) its training and test set.

Two publicly available hyperspectral image datasets (Fig 2) (available from: https://engineering.purdue.edu/~biehl/MultiSpec/hyperspectral.html) served as excellent testbeds to demonstrate the performance of the improved and optimized MDI algorithm for landcover classification, with a particular focus on vegetation cover. The first dataset was from the AVIRIS flight campaign in 1992 over Indian Pines in North-western Indiana [20]. The image size is 145 x 145 pixels with 220 spectral reflectance bands in the wavelength range of 400 nm to 2500 nm. A well-known ground truth data also comes with the image dataset with 10 identified classes: wheat, soybean-notill (no tillage), soybean-min (minimum tillage), soybean-clean till, grass/tree, grass/pasture, corn-notill (no tillage), corn-min (minimum tillage), woods, and hay. The spatial resolution is 20 m. The second dataset was a subscene of a 191-band Hyperspectral Digital Imagery Collection Experiment (HYDICE) Washington DC Mall image. Originally, there were 307 x 1208 pixels and 210 spectral bands covering the 400 nm to 2400 nm electromagnetic spectrum. We used an image size of 300 × 500 pixels with spatial resolution of approximately 3 m, to limit the analysis to the northern area with more varied land cover classes: water, grass, tree, road, and pathway. We added this test dataset to check on how the optimized indices discriminate between tree and grasses, specifically. The original dataset [21] included a thematic map with ground-truth labels. From the labeled data, we randomly sampled 30% as the training set and the rest as the test samples. The information classes and training and test samples for both images are listed in Table 1 and shown in Fig 1.

**Table 1:** The ground-truth classes of the AVIRIS and HYDICE datasets and the training and test sets used for the classes.

**AVIRIS**			
**Class**	**Train**	**Test**	**Total**
**Corn-min**	197	460	657
**Grass/tress**	166	387	553
**Corn-notill**	224	522	746
**Soybean-min**	369	860	1229
**Soybean-notill**	181	422	603
**Soybean-clean**	137	319	455
**Woods**	214	499	713
**Hay-windrowed**	96	225	321
**Wheat**	71	167	238
**Grass/pasture**	146	340	485
**HYDICE**			
**Class**	**Train**	**Test**	**Total**
**Water**	362	846	1208
**Tree**	317	741	1058
**Grass**	267	622	889
**Road**	193	451	644
**Pathway**	210	491	701

### Original moment distance

**Fig 3:**
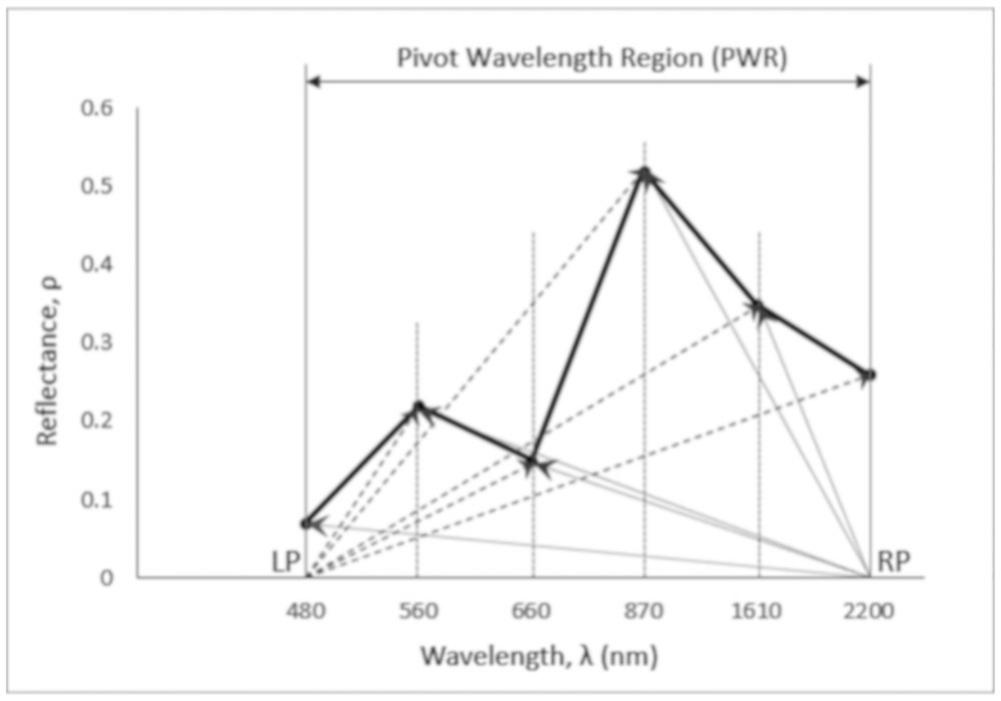
Schematic diagram of MDI applied on a sample spectral reflectance curve of a green vegetation (adapted from Salas and Henebry [12]). Note that the number of points between LP and RP pivots can vary depending on the number of bands analyzed or the width of the pivot wavelength region.

The moment distance (MD) framework (Fig 3) has two aspects: the set of equations that generate the metrics and the choice of positions within the reflectance curve to highlight. The MD framework that is described in the following set of equations and detailed in Salas and Henebry [12] [13], would generate the final MDI equation:

$${MD}_{LP}=\sum_{i=\lambda_{LP}}^{\lambda_{RP}}{\left({\rho_{i}}^{2}+{\left(i-\lambda_{LP}\right)}^{2}\right)}^{0.5}$$ (1)

$${MD}_{RP}=\sum_{i=\lambda_{RP}}^{\lambda_{LP}}{\left({\rho_{i}}^{2}+{\left(\lambda_{RP}-i\right)}^{2}\right)}^{0.5}$$ (2)

$$MDI={MD}_{RP}-{MD}_{LP}$$ (3)

where the moment distance from the left pivot (MD_LP_) is the sum of the hypotenuses constructed from the left pivot to the value at successively longer wavelengths (index *i*). In other words, MD_LP_ is the summation of hypotenuses from the wavelength location of left pivot (*λ*_LP_) to the wavelength location of right pivot (*λ*_RP_). For the hypotenuse, one base of the triangle is the difference from the left pivot (*i*-*λ*_LP_) along the abscissa and the other is simply the value of the reflectance (*ρ*) at *i* (Eq 1). Similarly, the moment distance from the right pivot (MD_RP_) is the sum of the hypotenuses constructed from the right pivot to the value at successively shorter wavelengths (index *i* from *λ*_RP_ to *λ*_LP_); where for the hypotenuse, one base of the triangle is the difference from the left pivot (*λ*_RP_-*i*) along the abscissa and the other is simply the value of the reflectance (*ρ*) at *i* (Eq 2). The final equation is the unbounded MDI (Eq 3).

### Optimized MD: Moment distance index normalized and moment distance ratio

The proposed optimized Moment Distance Index Normalized (MDIN) (Eq 4) shares the formal limitations of a normalized difference; however, it should be noted that loss of sensitivity so familiar to users of the NDVI would be much less of an issue in the MDIN. Unlike the strong spectral contrast that the NDVI exploits, the magnitude of the MD_LP_ is never too different from that of the MD_RP_. Thus, the value of the numerator is not dominated by a single pivot. More importantly, MDIN would address the boundless characteristic of MDI.

$$MDIN=\frac{{MD}_{RP}-{MD}_{LP}}{{MD}_{RP}+{MD}_{LP}}$$ (4)

Two other optimized MD metrics evaluated in this study include a simple Moment Distance Ratio (MDR) with the left pivot (LP) as numerator (Eq 5) and right pivot (RP) as numerator (Eq 6). Both MDRLR and MDRRL explain the true relationship between MDRP and MDLP and enhance the spectral differences between the moment distances derived from two opposing pivots.

$$MDRLR=\frac{{MD}_{LP}}{{MD}_{RP}}$$ (5)

$$MDRRL=\frac{{MD}_{RP}}{{MD}_{LP}}$$ (6)

### Other spectral indices and textural features

Apart from the original and optimized MDIs, we computed a set of narrow/broad-band spectral indices that have previously shown to perform well for image classification. We included NDVI, EVI, Normalized Difference Infrared Index (NDII) [22], Nitrogen Reflectance Index (NRI) [23], Carotenoid Reflectance Index (CRI) [9], Plant Senescence Reflectance Index (PSRI) [24], and Photochemical Reflectance Index (PRI) [25] as inputs in the classification to improve feature discrimination and accuracy of our target classes (Table 2). We calculated these broadband indices based on two or three spectral bands and selected them based on their application to monitor physiological stress in vegetation and their sensitivity to the presence of green foliar biomass. We averaged spectral bands to represent NIR (750–850 nm), red (600–700 nm), green (500–600 nm), and blue (400–500 nm) [12] for all broadband indices. For consistency, we used these same ranges of bands for both hyperspectral images in this study.

**Table 2:** Description of other spectral indices used as input predictor variables in this study.

Variables	Formula	Description/Application
**NDVI**	$\frac{Red-NIR}{Red+NIR}$	Exploits the strong differences in the red and NIR reflectance where contrast between vegetation and soil is maximal.
**EVI**	$2.5*\frac{NIR-Red}{1+NIR+6*Red-7.5*Blue}$	Effective in classifying high biomass regions like cultivated agricultural fields [29].
**NDII**	$\frac{\lambda 819-\lambda 1649}{\lambda 819+\lambda 1649}$	Used for sensitivity to water content.
**NRI**	$\frac{Green-Red}{Green+Red}$	Useful indicators for the estimation of biomass in crops [30].
**PSRI**	$\frac{Red-Blue}{NIR}$	
**PRI**	$\frac{\lambda 529-\lambda 580}{\lambda 529+\lambda 580}$	Best hyperspectral narrowband index for estimating crop evapotranspiration [31].

Image textures have shown in the past to be effective for landcover classification using very high resolution imagery [26] [27] [16] [28]. Here, we exploited the gray-level-gradient co-occurrence matrix analysis (GLGCM) to derive image textures: variance (VAR), entropy (ENT), correlation (COR), contrast (CON), and angular second moment (ASM). GLGCM measures use a gray-level spatial dependence matrix, which is a function of both the angular relationship and distance between two neighboring pixels. We implemented GLGCM on the three highest principal component (PC) score images that accounted for the most variances of all spectral bands. In total, we extracted 15 second-order statistical textural variables from the three highest PC scores in this study.

### Object-based image analysis

Object-based image analysis (OBIA) improves classification accuracy with respect to the traditional pixel-based approach. With OBIA, an object is represented in its true spatial landscape pattern instead of a squared classified pixel [32]. We produced image objects for all inputs used in the classification. Since object extraction is scale-dependent, we ran different scale levels during the initial segmentation process to find the best scale effects to incorporate on the prediction accuracy for hyperspectral images [33]. A high scale level could cause fewer defined segments, while a low scale level could result into over-segmentation [34]. Following Frohn et al. [35] and our initial results, we selected two scale levels 5 and 20 for AVIRIS and HYDICE, respectively. We implemented all GIS and remote sensing processes using ArcGIS v10.4 software [36] and the commercially-available GRASS GIS software [37].

### Random forest classifier

We used Random Forest (RF) [38] and compiled a number of codes in R [39] [40] for classification. RF is a non-parametric supervised classifier that uses Classification and Regression Tree (CART) through bagging, where it randomly picks a set of features and creates a classifier with a bootstrapped sample of the training data to grow a tree. With RF training data selection, it is possible that the same sample could be picked several times, whereas others may not be picked at all. Apart from RF being quite robust with highly collinear variables, the random selection process at each tree node causes low correlation among the trees and avoids over-fitting [41]. RF does not require assumption of the underlying distributions of the dataset input [42], making it a convenient method to use for hyperspectral images in the classification of invasive plants [43], flower species [44], landcover classes [45] [46], grass species [47], and crops such as wheat [30] and soybean varieties [18]. We generated decision trees following Colditz [48] and Reese et al. [49] that used the RF classifier on remotely sensed data.

We used all derived indices and textural variables as potential classification input variables in RF in order to find an ideal hyperplane that could discriminate landcover classes effectively. We also looked into the importance of each variable since RF is capable of measuring the importance of the individual input variable or a set of variables (e.g., spectral bands) in the classification. A high value of the normalized variable (which was based on the accuracies of the permuted out-of-bag samples, accuracies of the original samples, and the standard deviation) indicates that the variable has a high contribution for the entire RF. This capability of the RF further enhances the use of the classifier as a tool to combine with OBIA approach.

To thoroughly evaluate the performance of the modified algorithm to the classification, we ran different analyses using five sets of data with and without the moment distance metrics (Table 3). First, we ran the RF classifier without any MD metric (set 1). We then ran our R codes by including the MD metrics: all of set 1 plus MDIN (set 2), all of set 1 plus MDRLR (set 3), all of set 1 plus MDRRL (set 4), and all of set 1 plus original MDI (set 5). We determined model accuracies by creating and evaluating error metrics [50]: overall accuracy (OA) or the proportion correctly classified, producer’s accuracy (PA) or errors of omission (a feature is left out of the class being evaluated), user’s accuracy (UA) or errors of commission (a feature is incorrectly included in the class being evaluated), and kappa coefficient (measures the performance of the classification as compared to randomly assigning values).

**Table 3:** Five sets of data were separately used as inputs in the object-based random forest classification.

Sets	Variable Inputs	Total Segmented Variable Inputs
**1**	NDVI, EVI, NDII, NRI, PSRI, PRI, Texture (VAR, ENT, COR, CON, ASM)	21 segmented variables
**2**	NDVI, EVI, NDII, NRI, PSRI, PRI, MDIN, Texture (VAR, ENT, COR, CON, ASM)	22 segmented variables
**3**	NDVI, EVI, NDII, NRI, PSRI, PRI, MDRLR, Texture (VAR, ENT, COR, CON, ASM)	22 segmented variables
**4**	NDVI, EVI, NDII, NRI, PSRI, PRI, MDRRL, Texture (VAR, ENT, COR, CON, ASM)	22 segmented variables
**5**	NDVI, EVI, NDII, NRI, PSRI, PRI, Original MDI, Texture (VAR, ENT, COR, CON, ASM)	22 segmented variables

Note: MD metrics are underlined to highlight their inclusion in the dataset.

In addition, we presented McNemar’s test (Z) [51] to compare the classification results and to evaluate whether accounting for optimized MDI could improve results. If Z >0, then classifier 1 (e.g., with optimized MDI) is more accurate than classifier 2 (e.g., original MDI). The difference between classifiers 1 and 2 is statistically significant if |Z|>1.96.

## Results

### AVIRIS Indian Pines

**Fig 4:**
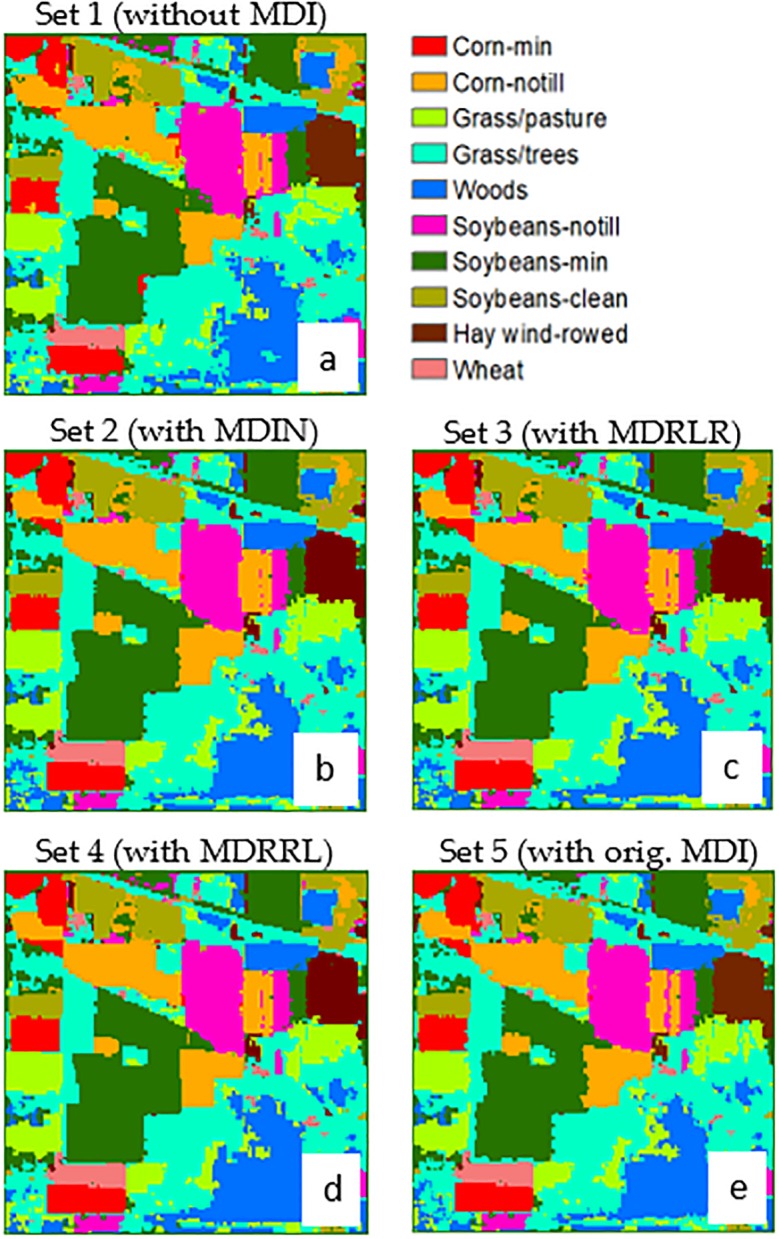
**Contrasting the classification results of using different datasets for AVIRIS Indian Pines image:** (A) dataset 1 without including any moment distance method, (B) dataset 2 with new MDIN, (C) dataset 3 with new MDRLR, (D) dataset 4 with new MDRRL, and (E) dataset 5 with original MDI. The maps were derived using object-based random forest classification.

Fig 4 shows the thematic maps produced using object-based RF classification algorithm. The estimated overall accuracy from the entire dataset with new optimized indices were 99% (Kappa = 0.98), 97% (Kappa = 0.96), and 95% (Kappa = 0.95) for MDRRL (set 4), MDIN (set 2), and MDRLR (set 3), respectively. The RF classifier did not perform better when the new optimized index was removed from the dataset (set 1), resulting in a 91% accuracy and Kappa = 0.90. With the original MDI (set 5), statistics resulted to an overall accuracy of 94% and Kappa = 0.93.

A comparison of class accuracies (Table 4) among five datasets showed that without the MDI, producer’s accuracy ranged from to 81% to 99% while user’s accuracies ranged from 71% to 99%. The largest source of error was soybean-min and corn-notill being classified as soybean-clean (confusion matrix, not shown). We observed misclassification between pasture and trees. Accuracies for set 1 were relatively lower compared to other sets for corn-min (PA = 81% and UA = 82%) and corn-notill (PA = 83% and UA = 86%). Set 1 also had the lowest UA for class grass/pasture (UA = 91.9). Our results showed that the absence of MDI in the training set resulted in a less effective discrimination between types of tillage (corn-min and corn-notill) and between vegetation classes (pasture, trees, woods). However, classification accuracies improved for corn-min (PA = 93% and UA = 90%), corn-notill (PA = 90% and UA = 88%), grass/pasture (PA = 98% and UA = 99%) when original MDI was introduced to the dataset (set 5).

**Table 4:** Summary of classification accuracies (%) from five sets of data using AVIRIS Indian Pines image: set 1 (no MDI), set 2 (with MDIN), set 3 (with MDRLR), set 4 (with MDRRL), and set 5 (with original MDI).

Class	Set 1 (no MDI)		Set 2 (MDIN ^b^)		Set 3 (MDRLR ^b^)		Set 4 (MDRRL ^a,b^)		Set 5 (orig. MDI ^b^)	
	PA	UA	PA	UA	PA	UA	PA	UA	PA	UA
**Corn-min**	81.1	82.0	96.2	93.0	98.1	95.6	99.0	97.4	93.0	89.6
**Corn-notill**	83.0	86.2	90.6	95.2	91.2	95.2	98.2	97.8	90.0	88.1
**Grass/pasture**	95.5	91.9	97.5	94.7	98.4	95.1	99.6	99.5	98.5	99.3
**Grass/trees**	98.0	98.9	99.6	97.9	98.7	98.8	100	98.4	98.8	97.9
**Woods**	98.8	99.4	100	100	100	100	100	100	99.7	100
**Soybean-notill**	98.2	89.4	99.1	97.4	98.8	96.8	98.9	98.8	99.6	94.6
**Soybean-min**	89.5	97.0	94.7	99.2	95.6	97.6	98.9	99.7	87.9	99.4
**Soybean-clean**	90.2	71.8	98.7	93.6	99.4	91.0	97.9	96.4	98.2	82.5
**Hay**	97.1	98.3	100	99.4	97.9	100	100	100	100	98.3
**Wheat**	99.7	98.1	96.7	100	100	91.2	100	100	100	96.5

The marks ^a,b^ signify that the set produces significant differences at the 5% level against set 5 and set 1, respectively.

The individual accuracies (PA and UA) for the optimized indices, MDIN (set 2) and MDRLR (set 3), were relatively high and ranged from 90% to 100%. Both sets produced comparable class accuracies and were slightly higher compared to the PA and UA of set 5 with original MDI, most especially for class corn-notill but not for grass/pasture. However, these increases in classification accuracies with MDIN and MDRLR did not result into statistically significant differences when compared to those obtained with original MDI.

MDRRL (set 4) was the only optimized method with a classification improvement considered statistically significant (Z = 2.54) over the one derived with original MDI (set 5) at 5% level. In Table 4, accuracies for corn-min and corn-notill have significantly improved with MDRRL, values ranged from 97% to 99%. For grass/pasture, grass/trees, woods, the accuracies ranged from 98% to 100%. Only set 4 with MDRRL has perfectly classified wheat crop and hay.

In terms of how the methods discriminated the ten classes, woods turned out to be best discriminated from other classes using MDRRL, MDRLR, and MDIN. MDRRL was also the best option in classifying wheat, minimizing classification confusion between corn-min and corn-notill, and between soybean-min and soybean-notill.

### HYDICE Washington DC Mall

**Fig 5:**
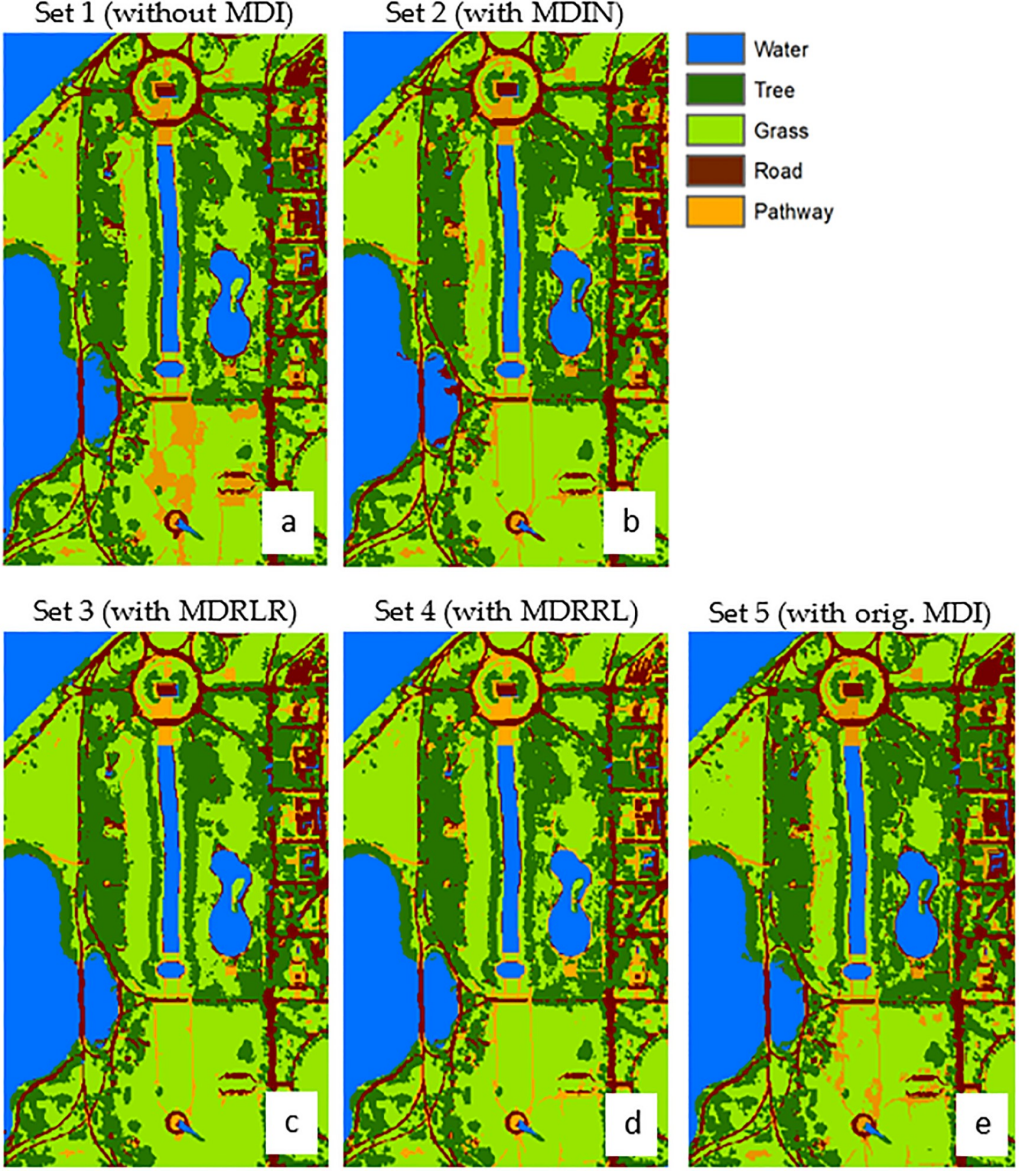
**Contrasting the classification results of using five datasets for HYDICE Washington DC Mall image:** (A) dataset 1 without including any moment distance method, (B) dataset 2 with new MDIN, (C) dataset 3 with new MDRLR, (D) dataset 4 with new MDRRL, and (E) dataset 5 with original MDI. The maps were derived using object-based random forest classification.

Fig 5 shows results of RF classifications from the five datasets for HYDICE Washington DC Mall image and Table 5 summarizes the overall accuracy for each class. The estimated overall accuracy for datasets with optimized indices were 99% (Kappa = 0.99) for MDRRL (set 4), 99% (Kappa = 0.99) for MDRLR (set 3), and 95% (Kappa = 0.95) for MDIN (set 2). Similar to the results for AVIRIS Indian Pines image, the RF classifier did not perform better when the optimized index was removed from the dataset (set 1), resulting in a lower accuracy (90%) and Kappa (0.90). The original MDI (set 5) statistics resulted in an overall accuracy of 95% and Kappa = 0.94, which is 4% lower than the best performing MDRRL.

**Table 5:** Summary of classification accuracies (%) from five sets of data using HYDICE Washington DC Mall image: set 1 (no MDI), set 2 (with MDIN), set 3 (with MDRLR), set 4 (with MDRRL), and set 5 (with original MDI).

Class	Set 1 (no MDI)		Set 2 (MDIN ^b^)		Set 3 (MDRLR^a,b^)		Set 4 (MDRRL^a,b^)		Set 5 (orig. MDI ^b^)	
	PA	UA	PA	UA	PA	UA	PA	UA	PA	UA
**Water**	100	100	100	100	100	100	100	100	100	100
**Tree**	86.6	90.7	90.6	99.8	99.8	99.8	99.8	100	91.5	96.7
**Grass**	90.5	94.7	94.8	96.6	100	99.9	100	99.9	100	94.4
**Road**	86.5	71.7	95.0	72.9	99.7	100	99.7	100	81.1	93.0
**Pathway**	78.5	66.0	97.1	97.6	100	98.7	100	99.8	86.3	100

The marks ^a,b^ signify that the set produces significant differences at the 5% level against set 5 and set 1, respectively.

Without MDI, producer’s accuracy ranged from to 78% to 100% while user’s accuracies ranged from 66% to 100%. The source of error for set 1 was the misclassification mainly between the classes pathway and road, and partly between the classes tree and grass. However, when we look at the other sets with moment distance algorithm, they not only improved the overall accuracy, but also enhanced the accuracy of each class.

In terms of sets with optimized algorithms, individual accuracies (PA and UA) were relatively high (> 90%), except for MDIN with UA = 73% for the class road. Set 3 (with MDRLR) and Set 4 (with MDRRL) provided the best results with significantly improved classifications. It could be seen in Table 4 that both MDRLR and MDRRL methods outperform the original MDI class by class, with 99% to 100% overall accuracy. For dataset with MDIN, improvements in classification were not statistically significant (at 5% level) when compared to the set results with original MDI. Among the five classes, water was easily identified regardless of the method used. Similar to results using AVIRIS Indian Pines dataset, trees turned out to be best discriminated from grass using MDRRL and MDRLR.

### Variable importance

Tables 6 and 7 lists the top 5 important variables according to object-based RF classification models for each dataset and image. For AVIRIS Indian Pines image, EVI seemed to be a predominant variable, ranking within the top three in 4 out of the 5 datasets (Table 6). Optimized MDIs—MDIN, MDRLR, and MDRRL—all ranked relatively higher on the list, with MDRRL being considered as the most important variable for set 4. Surprisingly, texture variables—VAR, ENT, COR, CON, and ASM—were only showing in the top 10 for all sets but set 4. Moreover, only one texture variable, entropy (ENT), appeared on set 3 with MDRLR as most important predictor. Among texture predictors, ASM led the list of importance for texture measures. The NDVI variable was listed at the bottom of the top 5 in all sets. Among other current indices, PRI was the most evident variable in the top 5 for 4 of the 5 sets.

**Table 6:** Rankings of the 5 object features with maximum importance across classes in the RF model using AVIRIS Indian Pines image.

Rank	Set 1 (no MDI)	Set 2 (MDIN)	Set 3 (MDRLR)	Set 4 (MDRRL)	Set 5 (original MDI)
	Variable	Variable	Variable	Variable	Variable
**1**	EVI	CON(PCA1)	MDRLR	MDRRL	EVI
**2**	ASM(PCA2)	MDIN	PRI	EVI	MDI
**3**	NDII	ASM(PCA1)	EVI	MDRRL	NDII
**4**	PRI	PRI	NDVI	NDVI	PRI
**5**	ENT(PCA1)	EVI	ENT(PCA1)	NRI	NDVI

The segmentation scale used was 5.

**Table 7:** Rankings of the 5 object features with maximum importance across classes in the RF model using HYDICE Washington DC Mall image.

Rank	Set 1 (no MDI)	Set 2 (MDIN)	Set 3 (MDRLR)	Set 4 (MDRRL)	Set 5 (original MDI)
	Variable	Variable	Variable	Variable	Variable
**1**	NDVI	NDVI	MDRLR	MDRRL	NDVI
**2**	ASM(PCA2)	MDIN	PSRI	NDVI	PSRI
**3**	PSRI	PSRI	NDVI	PSRI	MDI
**4**	ENT(PCA1)	ENT(PCA1)	PRI	NDII	ASM(PCA2)
**5**	NDII	ASM	ENT(PCA1)	PRI	NDII

The segmentation scale used was 20.

The HYDICE Washington DC Mall image classification showed NDVI as a variable with high importance for sets 1, 2, and 5 (Table 7). It also ranked within the top five for sets 3 and 4. EVI, which was a predominant variable for the AVIRIS Indian Pines image classification, did not appear in the top five. Optimized MDIs—MDIN, MDRLR, and MDRRL—all ranked relatively higher on the list, with MDRLR and MDRRL as the most important variables for sets 3 and 4, respectively.

## Discussion

The modified MDIs added another breadth of possibilities in the analysis of hyperspectral images. The results demonstrated the potential significant challenges in mapping and classifying landcover, specifically vegetation/crops and their management practices, using traditional approaches.

Our results offered more than merely a validation of the proposed optimized moment distance algorithms being tested. Results also identified what methods proved effective and what classification inputs were substantial. The inclusion of the optimized MDRRL in the classifications of AVIRIS and HYDICE showed significant differences and improvement in OA. The OA we observed was comparable to other studies that used the same datasets [52] [53]. However, the relatively high OA should be interpreted with some caution since it may not signify the true classification accuracy of the maps. For instance, a study with AVIRIS Indian Pines classification using support vector machine [54] had an OA = 94%, but individual class accuracies for some classes like soybeans-notill were relatively low (87%). The same thing was observed with our results for the same image dataset. For soybean-clean using set 5 (original MDI), the accuracy for the class was only 83% although the OA of the classification was 96%. The one promising result in this study was the use of the optimized MDRRL, where many classes for AVIRIS Indian Pines gained accuracies more than 95% or even 100% (OA = 98%). We found the same promising performance of the optimized MDRRL using the HYDICE Washington DC Mall image. These results established the robustness of our proposed MDRRL algorithm.

### Optimized indices on AVIRIS Indian Pines

**Fig 6:**
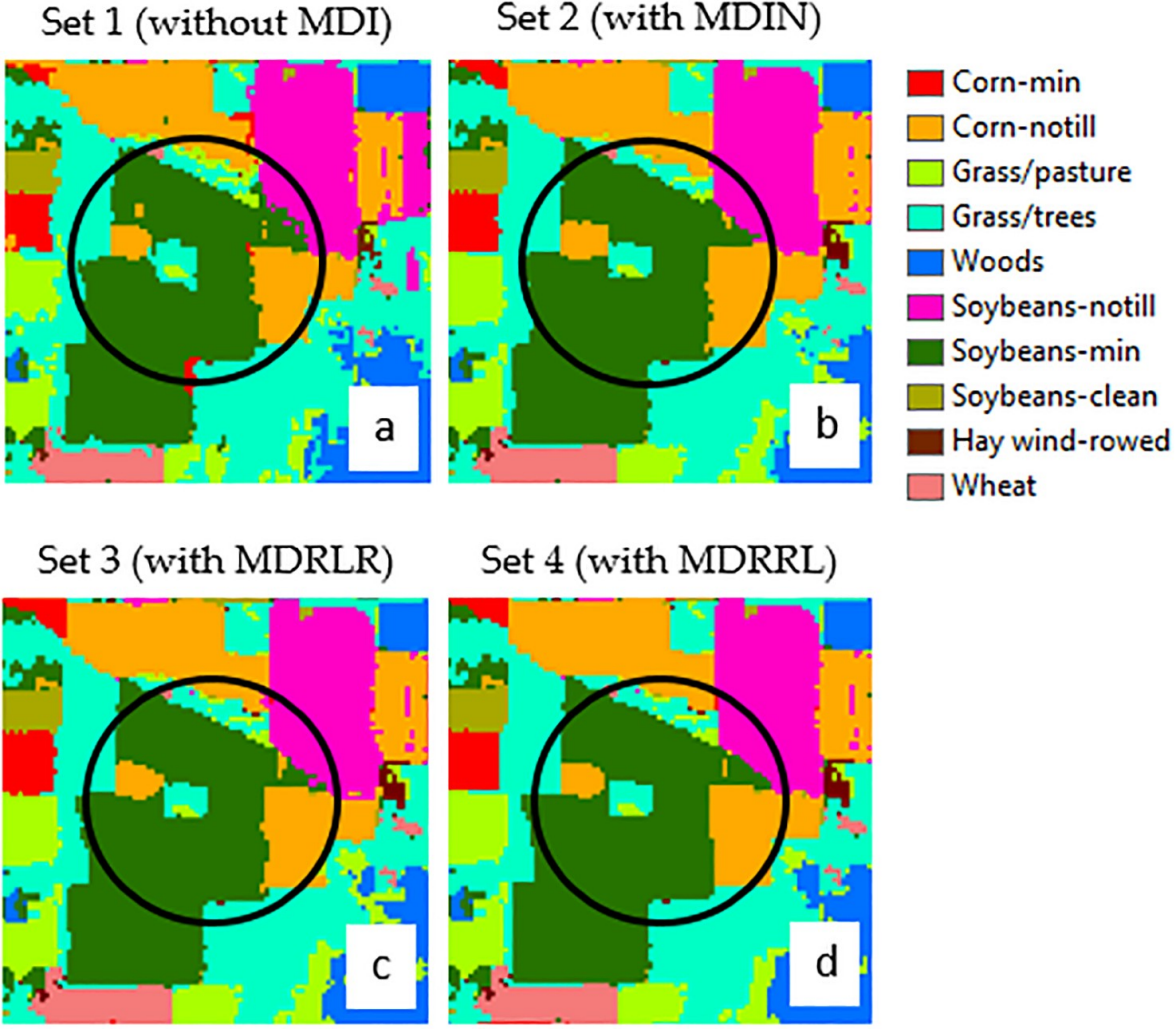
Encircled and magnified sample portion of AVIRIS Indian Pines classification maps, showing the difference of the performances of using a dataset (A) without MDI, (B) with MDIN, (C) with MDRLR, and (D) with MDRRL for classes soybean-min and corn-notill.

**Fig 7:**
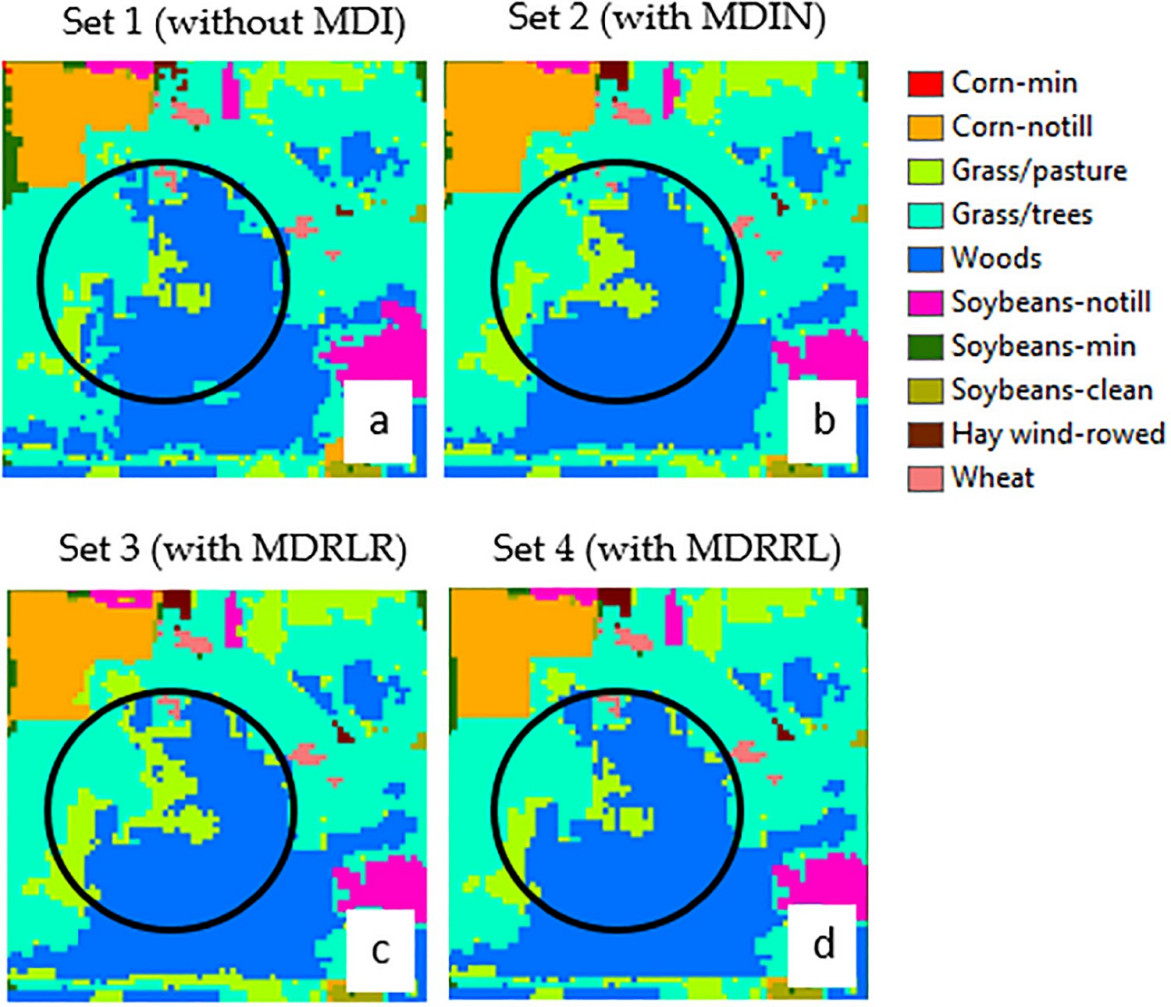
Encircled and magnified sample portion of AVIRIS Indian Pines classification maps, showing the difference of the performances of using a dataset (A) without MDI, (B) with MDIN, (C) with MDRLR, and (D) with MDRRL for classes grass/pasture and grass/trees.

Results obtained from datasets with MDIN, MDRLR, and without MDI displayed similar distributions and misclassifications particularly for soybean-min, corn-notill, grass/pasture, and grass/trees classes. However, against other algorithms tested here, the proposed MDRRL obtained the best and improved quantitative retrieval performance in discriminating between soybean-min and corn-notill, grass/pasture and grass/trees, and in classifying wheat—its clear advantage could be seen in Figs 6 and 7. When compared against other attempts that used AVIRIS Indian Pines for deep learning-based hyperspectral image (HSI) classification, our results are particularly remarkable in terms of accuracy. Class-wise, our results for MDRRL showed better individual accuracies for corn-notill and soybean-notill. Mapping methods such as the three HSI tests by Li et al. [28] and convolutional neural network applied to hyperspectral images by Paoletti et al. [55] both showed misclassifications for corn-notill as soybean-min, and for grass/pasture as grass/trees. Another study by Bhardwaj and Patra [52] that exploited genetic algorithms using full spectral features gave relatively high accuracies (94% to 99%) for corn-notill, soybean-min, grass/pasture, and grass/trees. The only constraint was that Bhardwaj and Patra [52] needed large filter parameters for constructing attribute profiles.

### Optimized indices on HYDICE Washington DC Mall

In HYDICE Washington DC Mall image, both datasets with MDRRL and MDRLR showed significant performance improvement over those with original MDI and without MDI. Classes discriminated using these datasets (sets 3 and 4) gained accuracies in the range of 98% to 100%. A study from Feng et al. [56] that used the same image, came out with relatively high overall classification accuracy (97%) for four classes (water, grass, tree, and road). However, upon inspection of individual class accuracies, confusion between grass and tree pixels was still evident that led to misclassifications.

### Performance of MDRRL on agricultural management system classification

**Fig 8:**
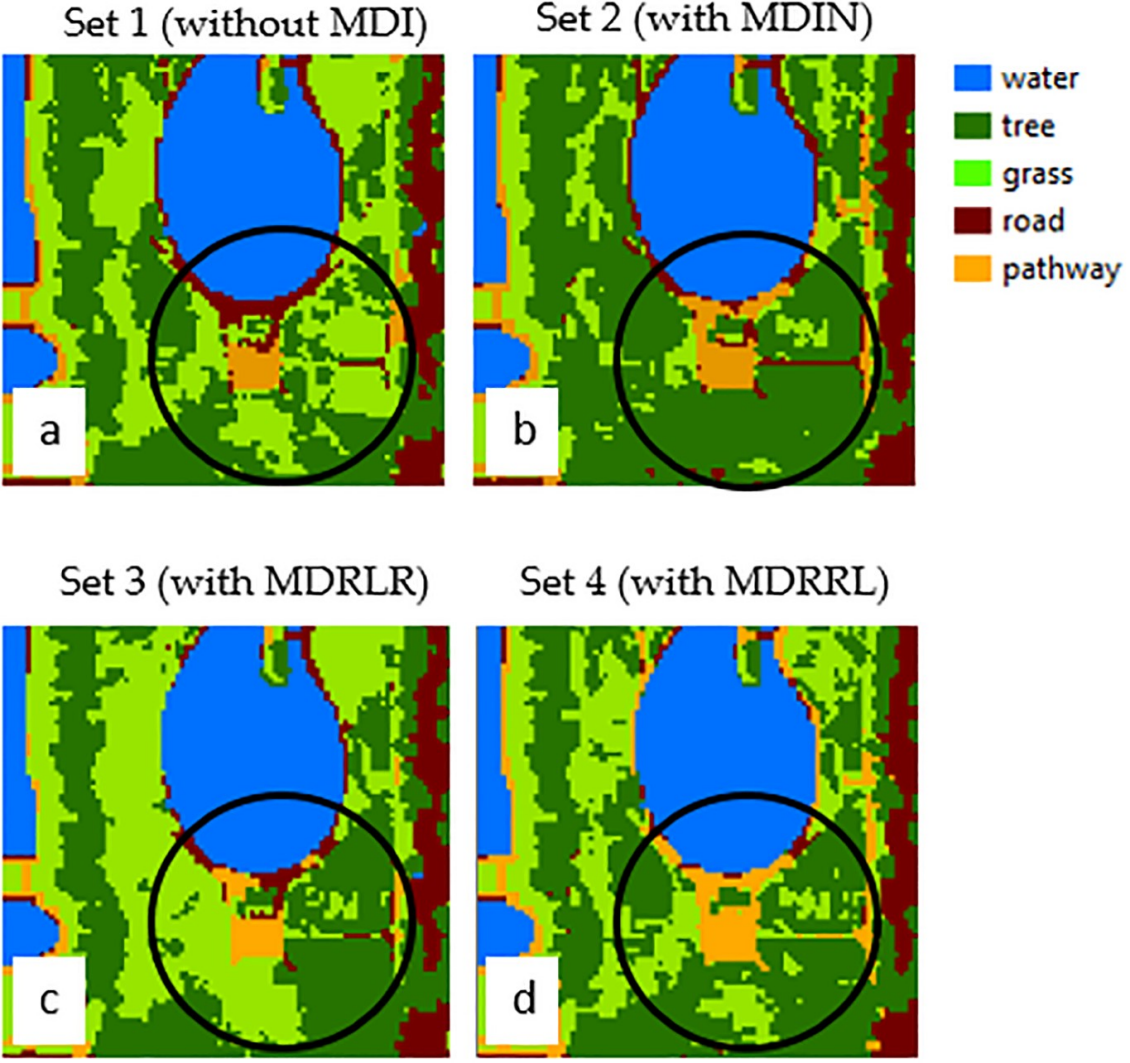
Encircled and magnified sample portion of HYDICE Washington DC Mall classification maps, showing the difference of the performances of using a dataset (A) without MDI, (B) with MDIN, (C) with MDRLR, and (D) with MDRRL for classes tree and grass.

**Fig 9:**
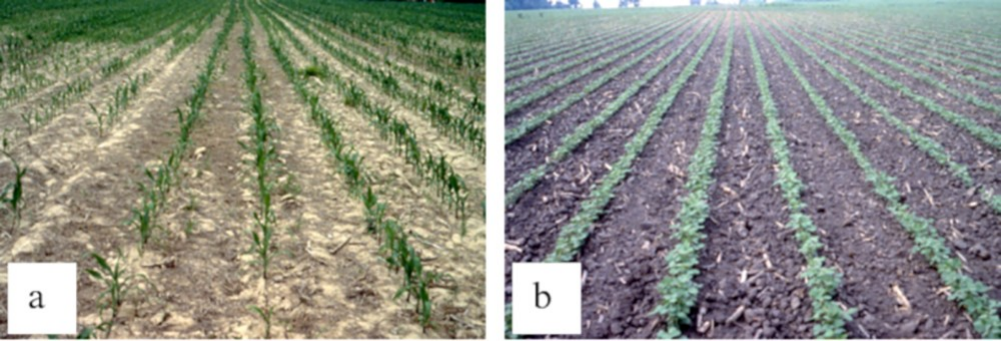
Sample ground reference photo for (A) corn-notill and (B) soybean-min taken at the AVIRIS image field site. Notill = no tillage; min = minimum tillage.

**Fig 10:**
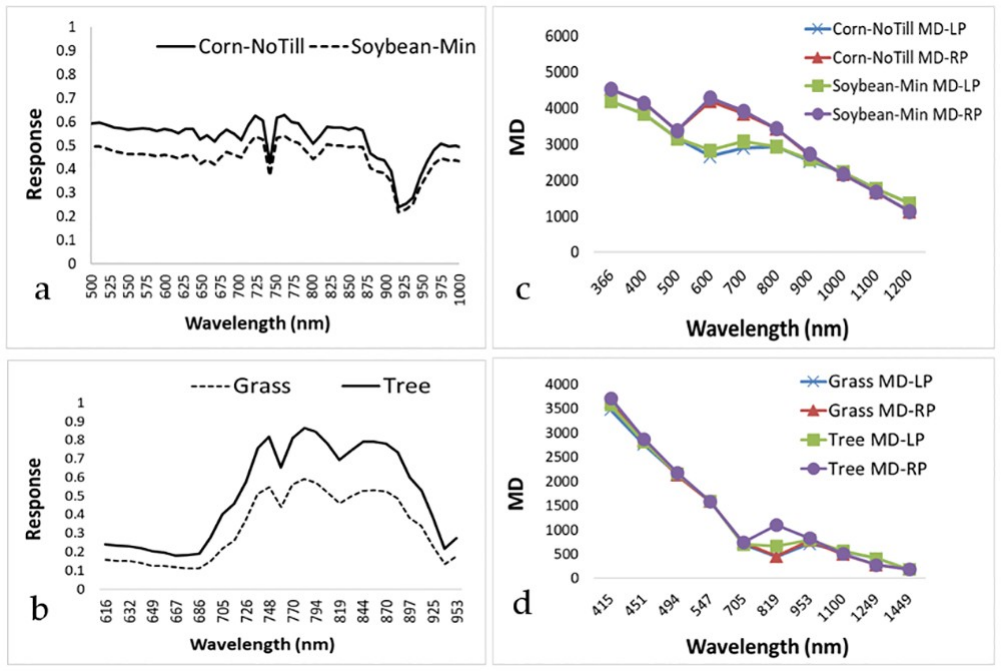
MD algorithm applied to the spectral responses of corn-notill and soybean-min for (A) AVIRIS, and grass and tree for (B) HYDICE, and how MDI values varied moving the pivot from left to right, and vice versa for (C) AVIRIS and (D) HYDICE images. Maximum values are observed at maximum shape differences, usually occurring at the inclusion of a curve peak or dip. Note that differences in curve shape could mean discrimination between classes.

The optimized MDRRL displayed superior worth in enhancing the image classifications with statistically significant overall accuracy improvements against the other algorithms. In both image datasets, MDRRL exhibited less scattered pixel problem (Fig 8), specifically for classes that could easily be misclassified due to their spectral similarities. In the following discussion, we looked at classes corn-notill and soybean-min from the AVIRIS image (Fig 9). To show the major influence of MDRRL, we averaged 20 random pixels from each class of corn-notill and soybean-min and manually computed the values of the optimized MDRRL from the generated curves (Fig 10). Notice the visually similar spectral characteristics of the two curves, especially their forms, such as locations of dips and peaks within the red and NIR regions (Fig 10A). Spectral indices, such as NDVI and EVI, could result into similar values for corn-notill and soybean-min when computed from these types of curves. But, for MDRRL, differences in absorption features were magnified to highlight the shape differences of each curve. To check, we fixed the RP at the right side of the curve (longer wavelength) and computed moment distances starting from the left (shorter wavelength) and moving forward to the right. We called the result MD-RP, or summation of moment distance from right pivot. We repeated the same procedure, but this time, we started in the opposite side. We fixed LP at the left side of the curve (shorter wavelength) and computed moment distances starting from the right (longer wavelength) and moving forward to the left. For this result, we called it MD-LP, or summation of moment distance from left pivot. Fig 10C showed the plots of the two summations. Clearly, differences in MD values were largest starting in the green region and moving towards the NIR. The divergences in MD values occurred at a wavelength range where curve shapes for corn-notill and soybean-min varied the most upon reaching 500 nm. Interpreted plainly, the difference between the two shapes corresponds to the unique spectral behaviors of corn-notill and soybean-min curves that were detected by MD. Maximum MD difference between corn-notill and soybean-min within 500 nm and 900 nm was higher for MD-RP (MD = 172) than MD-LP (MD = 94). With regards to ratio, MDRRL resulted in a value thrice larger than MDRLR (0.60 vs 0.20) when compared to corn-notill and soybean-min, which explains greater discrimination between the two classes. The difference in value between corn-notill and soybean-min reflected how the shape of the curve as viewed from reference RP varied from the one viewed from reference LP. With respect to classes grass and tree (Fig 10B) from the HYDICE Washington DC Mall image, we observed the same trend of the superiority of MDRRL over MDRLR. Differences in MD values showed the largest beginning in the red and towards the NIR regions (Fig 10D). These wavelength regions are also important for photosynthetic activity of plants [57]. Maximum MD difference between grass and tree within 705 nm and 953 nm was higher for MD-RP (MD = 650) than MD-LP (MD = 218). In terms of ratio, MDRRL resulted in a value twice larger than MDRLR (0.60 vs 0.30) when compared to grass and tree. We conclude that these minor differences in the absorption features detected by our method in the spectral shapes formed the basis for discriminating between tillage systems and landcover classes during classification.

### Important variables for mapping agricultural fields

While our analysis showed that the inclusion of the optimized MDI exhibited considerable improvement in the classification accuracy, there was no single object feature that dominated the variable importance during landcover mapping for both images. For AVIRIS Indian Pines data that was dominated by crop fields, the presence of EVI as a highly important predictor in the classification was justifiable. EVI has been effective in classifying regions with cultivated agricultural fields [29]. However, for the HYDICE Washington DC Mall image, EVI was not as effective as the other variables since the image was mostly composed of class grass and few patches of trees. All three optimized indices have the potential to be useful for image classification. However, among them, MDRRL and MDRLR showed the most potential. It ranked on top for both image classifications for sets 3 and 4, respectively. MDRRL reduced the overall confusion between classes grass and pasture for AVIRIS, and grass and trees for HYDICE. The less robust discrimination between these classes using the other optimized indices could be attributed to the shape of the spectral curves. We conclude that, although the classification accuracies for sets with MDIN were not at par with MDRRL and MDRLR, MDIN could be an important optical metric for classification of woods.

Overall, the advantage of the optimized MDIs against other spectral metrics could be summed up into three remarks. First, the optimized MDIs did not require us to select the best bands for mapping agricultural management systems to make them work, as they utilize the available bands of the AVIRIS and HYDICE products. It showed us the possibility of looking at and analyzing hyperspectral dataset in a different way. Second, all three algorithms characterized the untransformed shape of the spectral curve, such that a change of shape through the detection of minute peaks and troughs could mean distinction between classes. Third, the optimized MDIs could be unaffected by variance of soil reflectance [12] and could perform well in highly cultivated agricultural fields.

Textural features had lesser importance among variables when MDRRL and MDRLR were introduced into the classifications. However, we caution the complete exclusion of these features. Texture angular second moment (ASM) performed well in some sets and was also in the top 5. The use of all five image textures altogether may not be advisable since some of them have ranked with lesser importance. However, adding one or two in the classification, for instance ASM and ENT, could improve accuracy. Combining appropriate spectral indices, multivariate texture images, and a couple of optimized MDIs in the object-based RF classification algorithm, could lead into land use classes being accurately extracted.

With regards to the scale parameter in OBIA during our initial model runs, a finer coarse image segmentation scale (@5) was ideal for the AVIRIS image, while a much coarser image segmentation scales (@20) fit better for HYDICE image. A scale of 5 facilitated in differentiating the complex and much smaller patches of agricultural classes in AVIRIS, while a scale of 20 was sufficient to provide information on larger objects and more distinct classes in HYDICE. The results we found were consistent with the findings of other studies. For instance, the highest classification accuracy for agricultural land cover mapping was produced by a lower scale [58], guaranteeing high internal homogeneity in the segmented objects [59]. However, one of the constraints of our methodology was that, we did not incorporate a scale much finer than 5 or much coarser than 20. This could be one source of possible error in our analysis. Changing the scales could have effects on low performing optimized indices.

### Agricultural mapping implication of optimized MDIs

Although our main goal was to develop an improved shape index method, the results from this study presented an important implication for mapping agricultural management systems. Previous studies like that of Huggins and Reganold [60], Derpsch et al. [61], and Figuerola et al. [62] highlighted how sustainable agricultural management, such as no-tillage or minimum tillage, could play a vital role in reducing soil erosion and improving water quality, soil fertility and quality. There have been alternative spectral indices designed to map tillage [63] [64] based upon the 2100 nm cellulose absorption region. Nonetheless, authors of these indices found effects of variation of soil background and emerging green vegetation to be of critical concern. These concerns could be instigated by the fact that only two to three spectral bands of the hyperspectral data were chosen to design the indices.

The robustness of the optimized MDIs toward the use of all available bands of a hyperspectral image provides an exciting possibility and option for the identification of agricultural tillage practices. As opposed to other mapping methods [65][66] that may have lesser discriminative ability to differentiate spatial features between tillage systems, our approach of integrating the optimized spectral MDIs with other spatial features (e.g., textures) revealed minor spectral variance among different tillage environments. As we have shown, differences in absorption features from two tillage systems became magnified and highlighted the shape differences of each spectral curve. By not limiting the number of spectral bands, we demonstrated a viable strategy for agricultural tillage practice mapping that could easily discern a spectral response of one tillage system from another, thereby improving class separability.

## Conclusions

We developed and proposed a new and optimized moment distance index to improve the spatial-spectral classification of hyperspectral data for agricultural management systems. We conclude, based on our goal to obtain better classification accuracies not only for vegetation classes but for other landcover types, that it is worth integrating optimized MDIs for object-oriented classification of hyperspectral images. However, it is still unknown how optimized MDIs would perform when hyperspectral bands are limited, say for instance limiting the distance between LP and RP near the 2100 nm cellulose absorption region, which other existing indices had utilized in mapping tillage systems. This is something worth looking into in the future. One thing that is certain, however, that with proper selection of variables—spectral indices, textural variables, and optimized MDIs—we could obtain relatively high classification accuracies for individual landcover classes.

Our findings suggest that the use of object-based random forest classification, which effectively combines spectral information from input variables including optimized MDI, could allow the full potential of machine learning procedures for hyperspectral image classification. We highly recommend to conduct auxiliary studies on the uncertainties of object-based image classification, specifically applying our methods on various scale levels for different land features.
