# Correction: Modified shape index for object-based random forest image classification of agricultural systems using airborne hyperspectral datasets

**DOI:** 10.1371/journal.pone.0222474

**Published:** 2019-09-06

**Authors:** Eric Ariel L. Salas, Sakthi Kumaran Subburayalu

The are errors in the Funding statement. The correct Funding statement is as follows: This study was supported by USDA-NIFA 1890 Evans Allen Award, Grant Number NI181445XXXXG007 and NASA, Grant Number 80NSSC17K0653. The funders had no role in study design, data collection and analysis, decision to publish, or preparation of the manuscript.

## References

[1] Salas EAL, Subburayalu SK (2019) Modified shape index for object-based random forest image classification of agricultural systems using airborne hyperspectral datasets. PLoS ONE 14(3): e0213356 10.1371/journal.pone.0213356 30845216 PMC6405071

